# Hsp60 as a Novel Target in IBD Management: A Prospect

**DOI:** 10.3389/fphar.2019.00026

**Published:** 2019-02-08

**Authors:** Francesco Cappello, Margherita Mazzola, Abdo Jurjus, Marie-Noel Zeenny, Rosalyn Jurjus, Francesco Carini, Angelo Leone, Giuseppe Bonaventura, Giovanni Tomasello, Fabio Bucchieri, Everly Conway de Macario, Alberto J. L. Macario

**Affiliations:** ^1^Department of Experimental Biomedicine and Clinical Neuroscience University of Palermo (BIONEC-UniPA), Palermo, Italy; ^2^Euro-Mediterranean Institute of Science and Technology (IEMEST), Palermo, Italy; ^3^Department of Biology, College of Science and Technology, Temple University, Philadelphia, PA, United States; ^4^Department of Anatomy, Cell Biology and Physiology, American University of Beirut, Beirut, Lebanon; ^5^Department of Anatomy and Cell Biology, Faculty Development Associate for Education Research, Center for Faculty Excellence, The George Washington University School of Medicine and Health Sciences, Washington, DC, United States; ^6^Department of Microbiology and Immunology, School of Medicine, University of Maryland at Baltimore – Institute of Marine and Environmental Technology (IMET), Baltimore, MD, United States

**Keywords:** intestinal wall, microbiota, Hsp60, immune system, chaperoning system, inflammatory bowel disease, chaperonopathy, chaperonotherapy

## Abstract

Inflammatory bowel disease (IBD) encompasses various pathological conditions similar but distinct that share a multifactorial etiology, including involvement of the intestinal barrier function, the immune system, and intestinal microorganisms. Hsp60 is a chaperonin component of the chaperoning system, present in all cells and tissues, including the intestine. It plays important roles in cell physiology outside and inside mitochondria, its canonical place of residence. However, Hsp60 can also be pathogenic in many conditions, the Hsp60 chaperonopathies, possibly including IBD. The various clinico-pathological types of IBD have a complicated mix of causative factors, among which Hsp60 can be considered a putatively important driver of events and could play an etiopathogenic role. This possibility is discussed in this review. We also indicate that Hsp60 can be a biomarker useful in disease diagnosing and monitoring and, if found active in pathogenesis, should become a target for developing new therapies. The latter are particularly needed to alleviate patient suffering and to prevent complications, including colon cancer.

## The Bowel and Its Inflammatory Diseases: a Brief Overview

**FIGURE 1:**
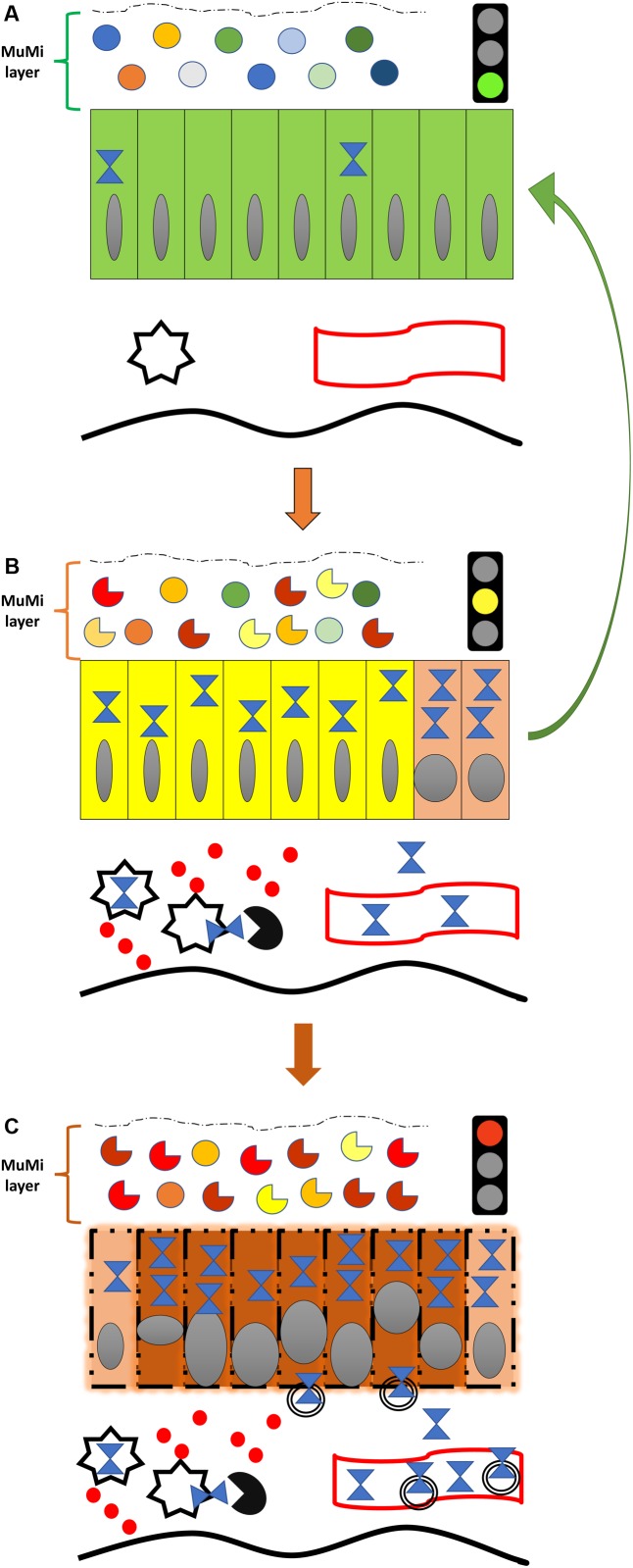
Diagram of the natural history of IBD from the standpoint of Hsp60 and its most feared complication, colorectal cancer. **(A)** Normal colon (or colon of an IBD patient in remission), showing the normal symbiotic flora (top icons; circles of various colors indicate different species of microbes of the normal, healthy microbiota) immersed in the mucous, both forming the functional mucous-microbiota (MuMi) layer, and epithelium with individual cells as rectangles (green) containing few Hsp60 molecules (blue hourglass). Also shown are immune system cells (star), e.g., macrophages and dendritic cells, and a vessel (blood or lymphatic; undulating band with red borders) in the lamina propria. The thick, orange vertical arrow pointing downward indicates initiation or relapse of IBD, illustrated in **(B)**. **(B)** Inflamed colon of an IBD patient in relapse. The flora is altered (dysbiosis; while circles indicate normal flora, see **(A)**, the incomplete circles of different colors indicate various microbes that are abnormal, not part of the healthy microbiota, and some may be pathogenic by themselves), and epithelial cells are changed (yellow) affected by the pathologic process with elevated levels of Hsp60, which may be involved in the initiation of carcinogenesis by inhibiting the apoptosis of epithelial cells with malignant DNA transformation, as represented by the two epithelial cells in orange (dysplasia; extreme right). Hsp60 is elevated also in immune system cells and is secreted into the extra-cellular space and/or exposed on the surface and stimulates T lymphocytes (black circle with quadrant missing) and secretion of pro-inflammatory cytokines (solid red circles). Secreted Hsp60 can also reach the general circulation as indicated by the hourglasses inside the lumen of the vessel. The thick vertical reddish arrow pointing downward indicates malignant transformation, illustrated in the **(C)**. The green arrow to the right suggests the expected effect (i.e., the reversal to a normal physiological situation) of IBD treatment aiming at inhibiting/blocking the anti-apoptotic and pro-inflammatory effects of Hsp60 (i.e., negative chaperonotherapy, consisting in blocking the pathogenic action of a chaperone). **(C)** Early (*in situ*) colon carcinoma developed on an IBD patient. The main feature is the profound transformation of many epithelial cells. Malignant cells are represented by reddish rectangles with altered nuclei, while cells still undergoing transformation (dysplasia) are shown in orange, as those shown in **(B)**, extreme right. Transformed cells have also elevated levels of Hsp60 and secrete the chaperonin into the extracellular space, mostly via exosomes (double-bordered circles). The amount of Hsp60 reaching the general circulation in exosomes or free increases considerably, and can be used as a biomarker for patient follow-up and for monitoring response to treatment. Note in the MuMi layer that the predominance of abnormal microbes (incomplete circles) with regard to the normal microbes (circles) is more marked than in **(B)**.

The intestinal tube is one of the first anatomical structures to form in the embryo, because of the immediate nutritional needs of embryonic cells, and from the intestinal tube other structures originate, such as exocrine and endocrine glands, airways, etc. (Chin et al., 2017). At the end of organogenesis, the intestinal wall is composed of multiple layers, classically described, from the inside, i.e., the lumen, to the outside, as mucosa, submucosa, *muscularis propria* and adventitia (or serosa, when the peritoneum is present). However, this description does not take into account the fact that, in living subjects, the most internal lining of the intestinal lumen consists of the mucus. This is produced by epithelial cells of the mucosa and contains about 100 billion microbial cells encompassing more than 10,000 different species, collectively called the intestinal microbiota. For this reason, we consider the most internal lining of the intestinal wall in the living organism to be the mucous-microbiota layer. We propose to name this layer “MuMi layer” on account of its two major components, namely the mucous and the microbiota, (Figure 1A). This functional layer has a loose (as compared with the other intestinal layers) and changeable structure mostly provided by the biofilms formed by bacteria, archaea, and micro-eukaryotes that constitute the microbiota. However, this layer is not visible under routine histologic examination; it is lost during the processing of the tissue for microscope observation due to the solubility of the mucous in alcoholic solutions. Consequently, this internal lining is systematically missed in histological studies and has, generally, been ignored or forgotten despite its key role in intestinal physiology and pathology.

The intestinal tube establishes multiple relationships with other anatomical districts both through visceral innervation and microvesicular trafficking. Examples of microvesicles are exosomes and outer membrane vesicles produced, respectively, by human and microbial cells. These vesicles can reach through the bloodstream virtually any anatomical districts, including some “protected sanctuaries” such as the brain, testicles, and thymus (Yáñez-Mó et al., 2015). In addition, intestinal mucosa cells produce and release soluble factors with autocrine- and paracrine-like properties with some reaching the general circulation and having systemic effects. This complex homeostasis is subverted in some intestinal pathological disorders, for example in inflammatory bowel disease (IBD).

Inflammatory bowel disease comprises chronic inflammatory pathologies of the intestinal tract, whose etiology is not yet fully understood (Shouval and Rufo, 2017). The two major types of IBD are ulcerative colitis (UC) and Crohn’s disease (CD). These two conditions share many clinical and pathological characteristics and are often considered together. However, they present different symptoms and clinical features, reflecting differences in the site of inflammation and in the type of immunological mechanisms involved in the pathogenesis that are characteristic of each of them (Gecse and Vermeire, 2018).

Inflammatory bowel diseases are considered multifactorial diseases triggered by an array of different factors, including immunological and intestinal barrier dysfunction as well as microorganisms (Nagao-Kitamoto et al., 2016; Bernstein and Forbes, 2017; Lanis et al., 2017; Shouval and Rufo, 2017; Gecse and Vermeire, 2018; Yu, 2018). Among these factors, the most prominent are: (i) imbalance of luminal mucosal homeostasis and induction of intestinal inflammation linked to environmental stimuli in genetically susceptible subjects; (ii) altered balance between regulatory mediators of inflammation, such as IFNγ and TNFα, that contribute to an inappropriate and sustained inflammatory response. Noteworthy, the high expression of pro-inflammatory cytokines and the increase of inflammatory cells can, in the long run, contribute to the formation of a microenvironment that supports the growth and proliferation of cancer cells and, for this reason, colorectal cancer (CRC) appears as one of the most serious complications of IBD; and (iii) loss of intestinal mucosal integrity. Consequently, intestinal dysbiosis occurs. This phenomenon is caused by the alteration in the composition of the intestinal microbiota due to exogenous (diet, smoking, antibiotics) and endogenous (psycho-physical stress) factors, Figure 1.

It is clear that IBDs have multiple etiopathogenic mechanisms. This complexity in etiology and pathogenic mechanisms, along with the need to design an optimal patient-customized therapy, generates an urgent need for exploring new alternatives. Here, we introduce the chaperonin Hsp60 as a candidate for investigation toward elucidating the pathogenic mechanisms of IBDs.

The reasons to focus on Hsp60 as biomarker useful in patient management and as a potential pathogenic agent in IBD are varied, as follows: (1) Hsp60 can induce production of pro-inflammatory cytokines (Sangiorgi et al., 2017; Cheong et al., 2018; Sun et al., 2018; Swaroop et al., 2018); (2) its quantities in UC mucosa vary in parallel with disease status, high in relapse and low in remission (Rodolico et al., 2010; Tomasello et al., 2011b); (3) its reported role in other conditions with inflammatory component, for instance atherosclerosis (Grundtman et al., 2011; Wick et al., 2014; Wick, 2016; Rahman et al., 2017); (4) there is ample epitope sharing between Hsp60s of various origins and human Hsp60 and other tissues (including intestinal ones). This molecular mimicry (Bachmaier and Penninger, 2005) elicits crossreactive antibodies reacting not-only with the immunizing antigen (e.g., Hsp60 from a bacterium infecting the intestinal or the genitourinary tract), but also with human Hsp60 and intestinal antigens (Füst et al., 2012). Autoimmunity due to molecular mimicry involving Hsp60 has been reported for various pathological conditions such as myasthenia gravis (Cappello et al., 2010; Marino Gammazza et al., 2012), Hashimoto’s thyroiditis (Marino Gammazza et al., 2014; Tonello et al., 2015), chlamydial infections (Campanella et al., 2009; Cappello et al., 2009), Guillain Barré syndrome (Loshaj-Shala et al., 2015), and periodontitis (Rizzo et al., 2012; Buhlin et al., 2015); and (5) despite all these revealing clues, research on the possible direct role of Hsp60 in the pathogenesis of IBD is not as abundant as it should be to make progress in disease monitoring and treatment.

In this short review, we aim to gather the few reports available on Hsp60 and IBDs and, thereby, provide a launching platform for innovative research to answer two key questions: (a) Does Hsp60 play a direct pathogenic role? and (b) If yes, What are the molecular mechanisms involved and where do they occur in the intestinal wall?

## Molecular Chaperones: Roles in Health and Disease

Molecular chaperones are essential for cell differentiation, tissue homeostasis, and organ remodeling in virtually all anatomical districts, including the intestinal tract (Rodolico et al., 2010; Tomasello et al., 2011b). They orchestrate the correct folding of many proteins and protect cells from the deleterious consequences of stress by preventing protein misfolding, premature degradation, or aggregation (Macario et al., 2013). The crucial role of molecular chaperones in intestinal tissues homeostasis is illustrated by the fact that their dysregulation in intestinal epithelial cells (including absorptive, Paneth’s, goblet, and entero-endocrine cells) drives colitis in experimental animals and patients with IBD (Ma et al., 2017). Specifically Hsp60, the focus of this review, plays a key role in the maintenance of intestinal epithelial stemness and proliferation (Berger et al., 2016), as we will discuss later.

Many chaperones are expressed in inflammation sites, probably participating in tissue regeneration (Hightower et al., 2000; Vitadello et al., 2010; Thanos et al., 2014). Also for this reason, in principle we consider inflammation a physiological phenomenon necessary for tissue repair through the proliferation and differentiation of normal cells but, when inflammation is either defective or excessive, it becomes pathogenic and leads to disease.

A set of proteins belonging to the class of molecular chaperones are heat shock proteins (Hsps), a group of proteins highly conserved during the evolution. The term “Hsp” derives from the observation that the concentration of these proteins increases following exposure to thermal stress (Ritossa, 1996). However, in the last decades it has been shown that Hsp levels can also increase after various stimuli other than thermal stress (Macario and Conway de Macario, 2005). They can be classified into two categories (Macario et al., 2013): (a) constitutive Hsps, are constitutively present inside the cell and act as molecular chaperones to guarantee the correct folding of other proteins, and the translocation of the mature proteins through the cell membranes, among other functions; and (b) inducible Hsps, that are produced, sometimes in large quantities, under stress conditions, whose primary role is to stabilize other proteins, preventing their denaturation during stress, and other functions unrelated to protein homeostasis.

Hsp chaperones typically reside in the various subcellular compartments such as nucleus, endoplasmic reticulum, microvesicles, and mitochondria (Macario and Conway de Macario, 2005), but can also occur extracellularly. For example, they can be secreted out of the cell via Golgi or in extracellular vesicles (EVs), such as exosomes (Campanella et al., 2012).

Extracellular vesicles are important for cell to cell communication and for this reason Hsps are now considered key proteins in intercellular cross-talk (Caruso Bavisotto et al., 2017). Once released, Hsps can act in two ways: (a) paracrine-like, when their targets are located near the cells that have secreted them, and (b) systemic (i.e., endocrine-like), when they reach their targets through the bloodstream.

Hsps play also an important role in carcinogenesis as many cancer-related proteins have been reported as Hsps clients (Rappa et al., 2012). Therefore, during carcinogenesis Hsps work for the tumor rather than for the host (chaperonopathy by mistake) (Macario et al., 2013). Consequently, cancer treatments targeting Hsps have been developed that can modulate various pathways of cancer progression, including neoplastic growth, angiogenesis, invasiveness, metastasis, and resistance to chemotherapy and radiotherapy (Rappa et al., 2012).

The role of Hsps in the immune response has been the focal point of a variety of studies (Macario et al., 2010). Hsps receptors have been identified on antigen-presenting cells (APCs) and a subset of T cells. It was hypothesized that Hsps are an important element in the defense against damage due to autoimmune mechanisms. Numerous studies have focused on the role of Hsps both as an element of pathophysiology in immune processes and as a potential therapeutic target. Another interesting aspect of the interaction between Hsps and immune system concerns the phenomenon of molecular mimicry (Bachmaier and Penninger, 2005; Cappello et al., 2009). This phenomenon is caused by a structural similarity between Hsp molecules present in pathogenic bacteria and human Hsps and other molecules. An immune response to a foreign Hsp of bacterial origin could result in cross-reaction against the host’s ortholog Hsp, generating an autoimmune condition. This mechanism could be the basis of sustained chronic inflammatory processes such as those that occur in IBD.

## Chaperonins, a Unique Class of Chaperones

Hsps are grouped considering their molecular weight into six groups: Super heavy Hsp, Hsp90, Hsp70, Hsp60 (chaperonins), Hsp40, and small Hsp or sHsp (kDa: 100 or higher, 81–99, 65–80, 55–64, 35–54, and 34 or lower, respectively) (Macario et al., 2013), and their nomenclature has been organized (Kampinga et al., 2009). Hsp60 belongs to the group falling within the 55–64 kDa range and has unique characteristics, for instance, it can form very large macromolecular complexes of about ∼ 1 MDa, and has been called “chaperonin” (Hemmingsen et al., 1988).

Classically, there are two groups of chaperonins (Vilasi et al., 2018), although a third group has recently been proposed (Rowland and Robb, 2017). Hsp60 belongs to Group I and it is classically considered a mitochondrial chaperone. Group II is represented by TRiC (TCP-1 Ring Complex), also called CCT (Chaperonin Containing TCP-1), and it works in the cytosol.

Although Hsp60 was initially considered an intramitochondrial protein, nowadays we know that it can also occur beyond mitochondria, for instance in the cytosol, the extracellular space, on the surface of normal and pathological cells (such as cancer cells), and other locales (Merendino et al., 2010). Its translocation to the extracellular medium is possible during stress conditions, such as inflammation and cancer (Figure 1B,C).

## Hsp60 in Bowel Physiology and Pathophysiology

Hsp60 is both constitutive and inducible. Under stress conditions its levels increase considerably, accumulates in the cytosol and can be secreted out of the cell via classic (Golgi’s) and alternative (exosomal) pathways (Merendino et al., 2010; Campanella et al., 2012).

Although Hsp60 can reside and function in a variety of intra- and extra-cellular locales, its canonical location is the mitochondrial matrix. Inside mitochondria, Hsp60 assists in the folding of all mitochondrial proteins, including those of the electron transfer chain for ATP production, thus playing a crucial role in the maintenance of cellular and tissue (including the intestinal tissue) physiology (Dencher et al., 2007; Takada et al., 2010; Berger et al., 2016). Absence of Hsp60 in intestinal epithelial cells causes the activation of the mitochondrial unfolded protein response (mtUPR) with mitochondrial dysfunction, resulting in loss of stemness and cell-proliferative capacity (Berger et al., 2016). Moreover, mitochondrial dysfunction is associated with paracrine release of WNT-related signals and hyperproliferation of residual stem cells that have escaped Hsp60 deletion (Berger et al., 2016). Hence, Hsp60 is crucial in the control of the epithelial stem cell niche of the bowel.

Upregulation of Hsp60 is a part of the exaggerated inflammatory response in some pathological conditions such as bronchitis (Cappello et al., 2011; Sangiorgi et al., 2017), keratoconjunctivitis (Leonardi et al., 2016), hepatitis (Barone et al., 2016), thyroiditis (Marino Gammazza et al., 2014), periodontitis (Rizzo et al., 2012), and IBD (Rodolico et al., 2010) (for details and illustrative images see Data Availability Statement just prior to the References list). We found Hsp60 in cells typical of inflammation in lamina propria in histological samples of IBDs but not in normal controls (Rodolico et al., 2010). This prompted us to hypothesize that this chaperonin could be implicated in the activation of the immune system leading to inflammation, particularly when alterations of the MuMi layer are present.

Subsequently, we reported that after treatment of patients with IBD with 5-aminosalicylic acid, alone or in combination with probiotics, amelioration of symptoms was associated with reduction of both inflammation and Hsp60. Interestingly, the levels of Hsp60 positively correlated with those of CD68-positive cells, and double immunofluorescence showed a high index of colocalization of the chaperonin and CD68 in lamina propria (Tomasello et al., 2011a). A correlation between gut microbiota imbalance and chaperoning system malfunction in IBD has also been suggested (Bellavia et al., 2013), as supported by data showing that probiotics supplementation reduce Hsp60 levels as well as its post-translational modifications (Barone et al., 2016); these modifications may be related to its secretion outside cells (Campanella et al., 2016), not only in its free, soluble form but also in the membrane of exosomes.

In agreement with these findings, antibodies against Hsp60 were found in patients with IBD (Elsaghier et al., 1992). These studies are worth expanding because of the potential implications of the findings in the development of therapeutic means for IBD.

Strong positivity staining for Hsp60 in mononuclear cells of the intestinal mucosa of patients with IBDs has been reported (Peetermans et al., 1995). Double staining for B7 and Hsp60 showed that Hsp60 was present in B7-positive cells, thus supporting the hypothesis that Hsp60 may play a role in the initiation and/or maintenance of the inflammatory process. Severe intestinal pathology was induced by the adoptive transfer of an Hsp60-specific CD8+ T-lymphocyte clone pre-activated by bacterial Hsp60, into TCR^-/-^ or SCID mice (Steinhoff et al., 1999). Colitis induction required the presentation of Hsp60 on MHC class I and depended on a functional role of TNF-α. In contrast to the findings obtained in other experimental models, inflammation did not depend on the presence of the resident bacterial flora in the MuMi layer. Thus, the results indicated that autoimmune Hsp60 CD8+ T cells, that were reactive to cellular Hsp60, mediated the pathogenesis of this very severe colitis. Furthermore, anti-inflammatory effects of prozumab, a humanized anti-HSP monoclonal antibody able to bind Hsp60, occurred in murine inflammatory colitis via: (a) induction of IL-10 secretion from naive human peripheral blood mononuclear cells; and (b) suppression of secretion of IFN-γ and IL-6 from anti-CD3-activated cells (Ulmansky et al., 2015).

Hsp60, in contrast to other molecular chaperones, is increased intracellularly in epithelial cells during early stages of colon carcinogenesis, i.e., intestinal adenomatous polyps with dysplasia (Cappello et al., 2003) (Figure 1B), a condition in which an alteration of the MuMi layer homeostasis is present (Kang and Martin, 2017). This information has been confirmed in other studies (Cappello et al., 2005; Campanella et al., 2015; Rappa et al., 2016) that correlated also Hsp60 levels to peritumoral inflammation and disease clinical course.

In tumor cells, Hsp60 binds pro-caspase 3, thus blocking apoptosis (Campanella et al., 2008). Hsp60 interacts also with cyclophilin D (Cyp-D), thus preventing Cyp-D dependent tumor-cell death through the formation of a complex with Hsp90 and tumor necrosis factor receptor-associated protein-1 (Ghosh et al., 2010). It has also been shown that Hsp60 plays a role in tumor cell survival through the activation of the NF-_K_B pathway (Chun et al., 2010). Hsp60 interacts directly with IKKα/β through the activation-dependent serine phosphorylation in a chaperone-independent manner to promote the TNF-α mediated activation of the IKKβ/NF-κB survival pathway (Chun et al., 2010). Finally, in tumor cells, Hsp60 is prone to undergo post-translational modifications that facilitate its secretion in the peritumoral microenvironment with pro-tumoral effects being likely (Gorska et al., 2013; Campanella et al., 2016; Marino Gammazza et al., 2017).

The data discussed in the preceding paragraphs implicate Hsp60 in the mechanisms causing various types of diseases, including IBD. Therefore, the need of more research becomes acutely apparent; research that should aim, for instance, at elucidating the molecular aspects of the Hsp60’s pathogenicity directly in the intestinal tissue. The results should help in the discovering of novel and efficacious therapeutic agents. In this regard, it is of interest to report that Hsp60 can be negatively modulated by specific inhibitors, both natural products and synthetic compounds (Pace et al., 2013; Cappello et al., 2014; Meng et al., 2018). These potential therapeutic agents, e.g., mizoribine, epolactaene, myrtucommulone, stephacidin B, avrainvillamide, o-carboranylphenoxyacetanilides, and gold (III) porphyrins were identified by chemoproteomics, and constitute the subject matter of another line of research that also deserves consideration in the efforts to cure IBD.

## Conclusion and Perspectives

Inflammatory bowel disease has a multifactorial etiopathogenesis. The molecular chaperone Hsp60 is emerging as a prominent player not only in the mechanisms of disease but also because of its potential as biomarker useful for diagnosis and patient monitoring and as therapeutic target. Normally, Hsp60 is one of the most important proteins for cell survival, proliferation, and differentiation. This chaperonin plays a key role in the maintenance of protein homeostasis inside mitochondria, including in the intestine. It also functions in many other processes unrelated to protein homeostasis beyond mitochondria. However, Hsp60 can be pathogenic. Of interest for this article are the potential roles of Hsp60 in IBD pathogenesis and complications, including carcinogenesis. Hsp60 has been implicated in IBD pathogenesis, in the initiation and/or maintenance of the inflammatory process. Furthermore, it is most likely involved in the process of bowel carcinogenesis in patients with IBD. Colon rectal cancer is one of the most serious complications of IBD, and Hsp60 seems to be involved in carcinogenesis. With this concept in mind, efforts should be made to elucidate whether or not Hsp60 plays a direct pathogenic role in IBD. If, indeed, Hsp60 does play a pathogenic role in IBD, research should be done to dissect the pertinent molecular mechanisms and to determine where exactly in the intestinal wall they occur. The results should help in the development of novel therapies targeting Hsp60.

In summary, the data discussed in this review support the notion that Hsp60 is worth investigating as a potential etiopathogenic factor in IBD. It is hoped that the results will provide the basis for IBD treatment focusing on the chaperonin to alleviate inflammation and prevent one of its most feared complications, colon cancer.

## Data Availability Statement

Supplementary bibliographic information and images concerning Hsp60 and chronic inflammatory diseases may be found online at: http://www.iemest.eu/en/the-chaperonopathies/28-the-chaperonopathies/224-picture-gallery.

## Author Contributions

All authors listed have made a substantial, direct and intellectual contribution to the work, and approved it for publication.

## Conflict of Interest Statement

The authors declare that the research was conducted in the absence of any commercial or financial relationships that could be construed as a potential conflict of interest.
